# Using e-mail recruitment and an online questionnaire to establish effect size: A worked example

**DOI:** 10.1186/1471-2288-11-89

**Published:** 2011-06-09

**Authors:** Helen M Kirkby, Sue Wilson, Melanie Calvert, Heather Draper

**Affiliations:** 1 MRC Midlands Hub for Trials Methodology Research (HTMR), School of Health and Population Science, University of Birmingham, Edgbaston, B15 2TT, UK

## Abstract

**Background:**

Sample size calculations require effect size estimations. Sometimes, effect size estimations and standard deviation may not be readily available, particularly if efficacy is unknown because the intervention is new or developing, or the trial targets a new population. In such cases, one way to estimate the effect size is to gather expert opinion. This paper reports the use of a simple strategy to gather expert opinion to estimate a suitable effect size to use in a sample size calculation.

**Methods:**

Researchers involved in the design and analysis of clinical trials were identified at the University of Birmingham and via the MRC Hubs for Trials Methodology Research. An email invited them to participate.

An online questionnaire was developed using the free online tool 'Survey Monkey^©^'. The questionnaire described an intervention, an electronic participant information sheet (e-PIS), which may increase recruitment rates to a trial. Respondents were asked how much they would need to see recruitment rates increased by, based on 90%. 70%, 50% and 30% baseline rates, (in a hypothetical study) before they would consider using an e-PIS in their research.

Analyses comprised simple descriptive statistics.

**Results:**

The invitation to participate was sent to 122 people; 7 responded to say they were not involved in trial design and could not complete the questionnaire, 64 attempted it, 26 failed to complete it. Thirty-eight people completed the questionnaire and were included in the analysis (response rate 33%; 38/115). Of those who completed the questionnaire 44.7% (17/38) were at the academic grade of research fellow 26.3% (10/38) senior research fellow, and 28.9% (11/38) professor. Dependent upon the baseline recruitment rates presented in the questionnaire, participants wanted recruitment rate to increase from 6.9% to 28.9% before they would consider using the intervention.

**Conclusions:**

This paper has shown that in situations where effect size estimations cannot be collected from previous research, opinions from researchers and trialists can be quickly and easily collected by conducting a simple study using email recruitment and an online questionnaire. The results collected from the survey were successfully used in sample size calculations for a PhD research study protocol.

## Background

Ideally, a study should be large enough to have a high probability (power) of detecting a statistically significant and clinically important difference [1] and sample size calculations are used to determine how large a study needs to be to achieve this [2-4]. For a simple sample size calculation to be made four values need to be known; the variance in the outcome, the effect size of interest, the level of significance and the power of the test [2,3].

In many studies, an estimate of effect size and standard deviation of the measurement may not be readily available, particularly if efficacy is unknown because it is a new or developing intervention, or if the trial is in a new target population. In situations such as these effect size can be estimated by gathering expert opinion on the likely effect size or necessary effect size to impact on clinical practice [5].

Two potential advantages of using email rather than traditional mail to conduct questionnaires are lower costs [6,7] and quicker data collection [8], but a disadvantage of this method is a potentially lower response rate [7,9-11].

The aim of this study was to assess the feasibility of using a simple email recruitment strategy and online questionnaire to produce an estimated effect size based upon expert opinion to inform sample size estimation for a randomised controlled trial.

## Methods

### Electronic Participant Information Sheet (e-PIS) study

A randomized controlled trial (RCT) is being developed at the University of Birmingham that aims to determine if an e-PIS (as compared to traditional paper based Patient Information Sheets), can improve recruitment to a study. The e-PIS differs from the more usual paper-based Patient Information Sheets in that it is available electronically (Internet-based) and gives potential research participants control/choice over the level and degree of detail of the information they access. An e-PIS has not been evaluated before, so no estimate of effect size (recruitment to the trial) exists to inform sample size estimation.

If the e-PIS, once developed, is to be used by researchers, its effect on recruitment rate needs to be sufficient to justify its additional cost. The effect size estimation from this questionnaire study will be used to calculate the sample size i.e. the number of participants needed for the e-PIS study to detect a statistically significant difference in recruitment rates.

### Development of the questionnaire

An online questionnaire was developed using the free online tool 'Survey Monkey^©^' [12] that allows development of a questionnaire with up to 10 questions and 100 responses without cost, and is ideal for conducting short, quick questionnaires of a relatively small sample. The questionnaire (appendix 1), designed specifically for this trial, aimed to estimate an effect size for the e-PIS study. The questionnaire briefly described the hypothetical e-PIS trial and then gave respondents a scenario in which an e-PIS that aimed to increase recruitment rates had been developed that cost approximately £1,000 (based on an estimated cost of development provided by the Medical Education Technology Team at the University of Birmingham). Participants were asked by how much they would need to see recruitment rates increase before they would consider using an e-PIS in their research, based upon expected baseline recruitment rates without using an e-PIS being 90%, 80%, 70%, 50% and 30% respectively. The likely recruitment rate for the e-PIS study was unknown, so the baseline recruitment rates aimed to cover a wide range of potential recruitment rates (30-90%).

### Study Participants

If the provision of an e-PIS increases recruitment rates, the findings are likely to be of relevance to any researcher undertaking human research. For this questionnaire study, therefore, any academic involved in such research was eligible to participate. This questionnaire study used a convenience study sample and included researchers involved in the design and analysis of clinical trials at the University of Birmingham and from the MRC Hubs for Trials Methodology Research (HTMR) [13]. The HTMR include trialists based in seven regional hubs throughout the UK with expertise in trials methodology research, and expertise in a range of areas such as improving patient recruitment and retention into trials, assessing new trial designs, and testing different approaches to data analysis. Researchers were invited to participate in the study by email with a URL link to the survey.

### Analysis

Analyses comprised simple descriptive statistics and were performed using Microsoft Excel Version 2007.

## Results

The invitation was sent to 122 people; 7 responded to say they were not involved in trial design and could not complete the questionnaire, 64 attempted it, 26 failed to complete it. Thirty-eight people completed the questionnaire and were included in the analysis giving a response rate of 33% (38/115).

Of the 26 participants who failed to complete the questionnaire, all exited before answering the scenario question (see Appendix 1 - Questionnaire).

Of those who completed the questionnaire 44.7% (17/38) were research fellows, statisticians or lecturers, 26.3% (10/38) were senior research fellows, senior statisticians or senior lecturers, and 28.9% (11/38) were professors or MRC Hubs for Trials Methodology Regional Directors.

The results demonstrate that on average, respondents wanted an e-PIS to increase baseline recruitment rates by between 6.9% and 28.9% before they would consider using it in their own studies (Table 1). The increase in recruitment rates sought was in adverse proportion to the baseline recruitment rates offered in the questionnaire. For example, for a baseline recruitment rate of 90%, participants wanted the e-PIS to improve recruitment rates by an average of 6.9% before they would consider using it, whereas for a baseline recruitment rate of 30% the average improvement required was 28.9%.

**Table 1:** Results from the online questionnaire

Expected consent rate without using the e-PIS	90%	80%	70%	50%	30%
**Mean percentage increase experts wanted**	6.9	10.7	13.5	20.2	28.9
**Standard deviation**	3.19	5.61	24.05	8.53	18.13
**Median percentage increase wanted**	9	10	11	20	25
**Inter quartile range**	5	5	10	17.5	30
**Range of responses seen**	0-10	0-20	0-30	0-50	0-70

At all baseline recruitment rates a smaller effect size was required for implementation of the e-PIS by more senior grades of staff (as compared with research fellows) (Table 2).

**Table 2:** Between group effect sizes (in %)

Baseline consent rate	Research fellow		Senior research fellow and above	
	Mean % (SD)	Median % (IQR)	Mean % (SD)	Median % (IQR)
**90%**	8.13 (2.59)	10 (5)	6.83 (3.46)	7.5 (5)
**80%**	12.5 (5.35)	10 (6.25)	9.85 (5.55)	10 (10)
**70%**	17.5 (8.12)	17.5 (11.25)	11.39 (5.57)	10 (4.375)
**50%**	28.08 (9.02)	25 (5)	13.45 (7.82)	10 (10)
**30%**	37.14 (16.95)	30 (15.08)	19.63 (13.50)	20 (20)

## Discussion

This study demonstrates that email recruitment and an online survey provide a rapid method to obtain a meaningful estimate of effect size and associated variability, which can be used to inform sample size calculations. Whilst the example in this paper shows how the methodology could be used to establish an effect size based on an increase in recruitment rates, it could be easily adapted to suit other studies. For example, for a study that tests the effectiveness of an intervention to increase vaccination rates it is reasonable to expect that the intervention would only be used outside of a research environment if it increased vaccination rates sufficiently. The questionnaire (appendix 1) could be adapted to ask participants how much the intervention would need to increase the vaccination rate by before they would implement the intervention in their area. The result would still be a meaningful effect size that could be used in a sample size calculation.

The results of the questionnaire demonstrate that for this scenario (increasing recruitment rates), even though the cost of the proposed e-PIS was relatively low, participants required a larger increase in recruitment rates when the baseline recruitment rate was low. This may reflect researchers perceptions on acceptable response rates required for validity and generalisabity.

As seen in other Internet based questionnaire studies [7,9-11], response rate was low (33%). The study sample, however, included academics at various stages of their career with relevant trials experience. Notably, over half of the participants were senior academics with extensive experience in trial design and analysis.

Twenty-six participants started but failed to complete the questionnaire, and since questionnaire responses were anonymous and no follow up of participants was conducted, reasons for non-completion could not be collected. It may be that once they started they realised they did not have experience required to provide answers, they did not understand the questions, or they became distracted and because it was online rather than a paper questionnaire on their desk, they forgot to go back to complete it. Participants also may have accidentally closed their browser before submitting the completed questionnaire meaning they would have lost their answers up to that point and did not want to complete it again. This is a further potential problem not encountered with paper questionnaires. If this questionnaire study were to be adapted for use in other studies it would be useful to collect feedback from participants.

Three grades of academics completed the questionnaire: research fellows, senior research fellows, and professors. Lower grades of academics tended to want the e-PIS to have a greater impact on consent rate before they would consider using it. For example, for a common baseline consent rate of 70%, only 8.3% of research fellows wanted to see an increase of below 5%, whereas 16.7% of academics above the level of senior research fellow would have accepted an increase of below 5%. At the other end of the scale, 25% of research fellows wanted to see an increase of above 20%, whereas no academics above senior research fellow level required an increase in recruitment rates above 20% in order for them to use an e-PIS. Whilst this small questionnaire study was not powered to evaluate between group differences, these exploratory analyses show that different levels of academics may have different criteria for deciding whether or not to use an e-PIS in their research and therefore want different effect sizes.

### Limitations

We utilised a convenience sample of participants with a broad range of research expertise, and this may have introduced bias. The observed variability in responses by different grades of academic staff illustrates the need, for future applications of this method of estimating effect sizes, to carefully consider the sampling frame and to use random sampling to improve the generalisability of results.

If the methodology described in this paper were to be used in other studies, a questionnaire specific to that study would need to be developed that took into account the study's estimated recruitment rate and related costs. This paper aimed to describe the methodology of using email recruitment and an online questionnaire to estimate effect size, and did not aim to produce a validated questionnaire for use in other studies.

## Conclusions

This paper has shown that in situations where effect size estimations cannot be collected from previous research, opinions from researchers and trialists could be quickly and easily collected by conducting a simple study using email recruitment and an online questionnaire. The results collected from the survey were successfully used in sample size calculations for a PhD research study protocol. Nevertheless, this worked example was restricted to one research study and further evidence is required to determine the application of the methodology to other studies.

## Competing interests

The authors declare that they have no competing interests.

## Authors' contributions

HK conceived and designed the research, collected data, made substantial contribution to the analysis and interpretation of the data and drafted the manuscript.

MC, SW and HD helped to design the research, made substantial contribution to the analysis and interpretation of the data and revised the manuscript for important intellectual content. All authors have read and approved the final manuscript.

## Pre-publication history

The pre-publication history for this paper can be accessed here:

http://www.biomedcentral.com/1471-2288/11/89/prepub

## Appendix 1 - Questionnaire

Job Description

Job title .......................................................................

Role in research design ...........................................................................................................................................

Which sector do you work in? ................................................................................................................................................

**Scenario: **Imagine that an Internet based electronic patient information sheet (e-PIS) has been shown to improve patient recruitment into a trial. There is a cost of around £1000 to develop and host e-PIS for each study.

Thank you for taking the time to complete this questionnaire. Answers will be used to establish an effect size and carry out sample size calculations for a PhD project.

By how much would you need to see patient recruitment increase by before you would consider using e-PIS in your study?

1. If the expected patient recruitment using the standard paper patient information sheet is 90% of patients contacted?.......... %

2. If the expected patient recruitment using the standard paper patient information sheet is 80% .......... %

3. If the expected patient recruitment using the standard paper patient information sheet is 70% .......... %

4. If the expected patient recruitment using the standard paper patient information sheet is 50% .......... %

5. If the expected patient recruitment using the standard paper patient information sheet is 30% .......... %

*Thank you*

That is the end of the questionnaire, thank you again for your time.
